# Use of Net Reclassification Improvement (NRI) Method Confirms The Utility of Combined Genetic Risk Score to Predict Type 2 Diabetes

**DOI:** 10.1371/journal.pone.0083093

**Published:** 2013-12-20

**Authors:** Claudia H. T. Tam, Janice S. K. Ho, Ying Wang, Vincent K. L. Lam, Heung Man Lee, Guozhi Jiang, Eric S. H. Lau, Alice P. S. Kong, Xiaodan Fan, Jean L. F. Woo, Stephen K. W. Tsui, Maggie C. Y. Ng, Wing Yee So, Juliana C. N. Chan, Ronald C. W. Ma

**Affiliations:** 1 Department of Medicine and Therapeutics, The Chinese University of Hong Kong, Hong Kong SAR, China; 2 Hong Kong Institute of Diabetes and Obesity, The Chinese University of Hong Kong, Hong Kong SAR, China; 3 Li Ka Shing Institute of Health Sciences, The Chinese University of Hong Kong, Hong Kong SAR, China; 4 Department of Statistics, The Chinese University of Hong Kong, Hong Kong SAR, China; 5 School of Biomedical Sciences, The Chinese University of Hong Kong, Hong Kong SAR, China; 6 Center for Genomics and Personalized Medicine Research, Center for Diabetes Research, Wake Forest School of Medicine, Winston-Salem, North Carolina, United States of America; Innsbruck Medical University, Austria

## Abstract

**Background:**

Recent genome-wide association studies (GWAS) identified more than 70 novel loci for type 2 diabetes (T2D), some of which have been widely replicated in Asian populations. In this study, we investigated their individual and combined effects on T2D in a Chinese population.

**Methodology:**

We selected 14 single nucleotide polymorphisms (SNPs) in T2D genes relating to beta-cell function validated in Asian populations and genotyped them in 5882 Chinese T2D patients and 2569 healthy controls. A combined genetic score (CGS) was calculated by summing up the number of risk alleles or weighted by the effect size for each SNP under an additive genetic model. We tested for associations by either logistic or linear regression analysis for T2D and quantitative traits, respectively. The contribution of the CGS for predicting T2D risk was evaluated by receiver operating characteristic (ROC) analysis and net reclassification improvement (NRI).

**Results:**

We observed consistent and significant associations of *IGF2BP2*, *WFS1*, *CDKAL1*, *SLC30A8*, *CDKN2A/B*, *HHEX*, *TCF7L2* and *KCNQ1* (8.5×10^−18^<*P*<8.5×10^−3^), as well as nominal associations of *NOTCH2*, *JAZF1*, *KCNJ11* and *HNF1B* (0.05<*P*<0.1) with T2D risk, which yielded odds ratios ranging from 1.07 to 2.09. The 8 significant SNPs exhibited joint effect on increasing T2D risk, fasting plasma glucose and use of insulin therapy as well as reducing HOMA-β, BMI, waist circumference and younger age of diagnosis of T2D. The addition of CGS marginally increased AUC (2%) but significantly improved the predictive ability on T2D risk by 11.2% and 11.3% for unweighted and weighted CGS, respectively using the NRI approach (*P*<0.001).

**Conclusion:**

In a Chinese population, the use of a CGS of 8 SNPs modestly but significantly improved its discriminative ability to predict T2D above and beyond that attributed to clinical risk factors (sex, age and BMI).

## Introduction

Type 2 diabetes (T2D) is one of the most common chronic diseases characterized by insulin resistance and relative insulin deficiency [1]. The number of people with T2D was estimated to increase from 285 million adults in 2010 to 439 million adults by 2030, posing an enormous strain to healthcare systems worldwide [2].

The development of T2D is caused by interplay between multiple genetic variants, lifestyle and environmental factors. In the Framingham Offspring Study, a simple clinical model including parental history of T2D, body mass index (BMI), high density lipoprotein cholesterol (HDL), triglycerides (TG), blood pressure (BP) and fasting plasma glucose (FPG) predicted T2D risk [3]. However, family history alone containing both genetic and shared environmental information, has low predictive power in clinical diagnosis [4] since each family member can differ genetically.

With the high-throughput genotyping technologies, genome-wide association studies (GWAS) not only confirmed the candidate genes such as *PPARG* [5], *KCNJ11* [6], *TCF7L2* [7] and *WFS1* [8], but also identified more than 70 novel loci for T2D risk [9], [10], [11], [12], [13], [14], [15], [16], [17], [18], [19], [20]. The majority of these variants conferred T2D risk through pancreatic beta-cell dysfunction [17], [21], [22], while only a few like *PPARG*, *FTO* and *IRS1* affected fat metabolism [12], [17], [22]. The use of a Combined Genetic Score (by summing up the number of risk alleles of these diabetes loci; CGS) has been shown to predict T2D risk better than using each genetic loci alone [23], [24], [25], [26], [27], [28], [29], [30], [31], [32], [33], [34], [35]. Other groups have demonstrated that pathway-specific CGS, constructed by using beta-cell function-related loci, was associated with reduced beta-cell function [24], [36], [37], [38], [39]. Despite these advancements in our understanding of the T2D genetics, the discriminative power of GCS above and beyond clinical risk factors remains low. In the present study, we investigated the individual and combined effects of 14 loci relating to beta-cell function in predicting 1) risk of T2D in a case-control cohort; 2) glucose-related traits in healthy subjects; 3) clinical characteristics in T2D patients, and 4) use of insulin in a Chinese population. We also used both receiver operating characteristic (ROC) analysis and net reclassification improvement (NRI) to assess the contribution of the CGS in predicting T2D risk.

## Research Design and Methods

### Ethics Statement

Written informed consent was obtained from all participants or parents of adolescents as appropriate. This study was approved by the Clinical Research Ethics Committee of the Chinese University of Hong Kong.

### Subjects

Details of the study design, ascertainment, inclusion criteria and phenotyping procedures of subjects have been reported [32], [40], [41]. All study subjects were of southern Han Chinese ancestry residing in Hong Kong. The case cohort consisted of 5882 unrelated T2D patients (mean age 56.8±13.3 years, 46% male, mean duration of T2D 7.1±6.7 years) selected from the Hong Kong Diabetes Registry (HKDR) [42]. The HKDR was established as a quality improvement program at the Prince of Wales Hospital since 1995. We made use of the universal health care system which provides more than 95% of chronic care to patients in Hong Kong. Once a diabetic subject is enrolled, he or she will be observed until death. Subjects in the cohort include patients referred from primary care clinics for complications assessment, as well as patients from specialist clinics. Subjects in the case cohort from HKDR were enrolled between 1995 and 2005. Around 46% of these patients had BMI≥25 kg/m^2^, consistent with the general characteristics of type 2 diabetes patients in our locality. T2D was diagnosed according to the 1998 World Health Organization (WHO) criteria. Type 1 diabetic patients with acute ketotic presentation, or patients with non-Chinese or unknown nationality, or missing data on type of diabetes, or continuous requirement of insulin within 1 year of diagnosis were excluded. The healthy control cohort consisted of 2569 subjects ascertained from 3 sources: a) 1057 adolescents (mean age 15.3±1.9 years, 46% male) from a community-based school survey, b) 586 adults (mean age 41.3±10.5 years, 45% male), and c) 926 elderly (mean age 72.3±5.3 years, 51% male) from two community-based health screening programs. To obtain a representative sample population of Hong Kong Chinese adolescents, we randomly selected schools and students using a computer-generated coding system. Those with chronic illnesses such as diabetes with or without drugs were excluded from the study [43]. Adults recruited from a territory-wide health awareness and promotion program were randomly selected by stratified random sampling with computer-generated codes in accordance to the distribution of occupational groups [44]. The elderly were recruited from community centers for the elderly and housing estates in Hong Kong since 2001. By using the stratified sampling technique, approximately one third of participants were randomly selected from each of the following age groups: 65–69, 70–74, and ≥75 years old [41]. The clinical characteristics of subjects in case and control cohorts are summarized in Table 1.

**Table 1:** Clinical and metabolic characteristics of 5882 subjects with type 2 diabetes (T2D) and 2569 healthy controls in Chinese population.

Characteristics	Healthy Adolescents	Healthy Adults	Healthy Elderly	T2D subjects
N (male%)	1057 (45.6%)	586 (45.1%)	926 (51.2%)	5882 (45.5%)
Age (years)	15.3±1.9	41.3±10.5	72.3±5.3	56.8±13.3
Age-at-diagnosis (year)	–	–	–	49.7±12.6
Body mass index (kg/m^2^)	19.9±3.6	22.9±3.3	23.2±3.3	25.1±3.9
Fasting plasma glucose (mmol/l)	4.7±0.4	4.8±0.4	–	–
Fasting plasma insulin (pmol/l)	45.1 (35.5–60.5)	41.1 (26.0–58.3)	–	–

Data are shown as N, %, mean±SD or median (interquartile range).

### Clinical Studies

All participants were examined in the morning after an overnight fast. Anthropometric measurements including waist circumference (WC), body weight and height were documented. Body mass index (BMI) was calculated as weight (kg) divided by squared height (m^2^). Central obesity was defined as WC≥90 cm for male or ≥80 cm for female. Fasting blood samples were collected for DNA extraction and measurements of hemoglobin A_1c_ (HbA_1c_), fasting plasma glucose (FPG) and insulin (FPI). Homeostasis model assessment of insulin resistance (HOMA-IR) was calculated as (FPI [mU/l]×FPG [mmol/l])÷22.5, and homeostasis model assessment of beta-cell function (HOMA-β) was calculated as FPI×20÷(FPG−3.5) [45]. Glomerular filtration rate (eGFR) was estimated using the abbreviated Modification of Diet in Renal Disease (MDRD) formula further adjusted for the Chinese ethnicity: eGFR = 186×[S_CR_×0.011]^−1.154^×[age]^−0.203^×[0.742 if female]×[1.233 if Chinese] where S_CR_ is serum creatinine expressed as µmol/l and 1.233 is the adjusting coefficient for Chinese population [46]. Use of medications, including oral blood glucose-lowering agents and insulin, were also recorded for all T2D patients. Anti-hypertensive medications included all blood pressure lowering drugs except for angiotensin converting enzyme (ACE) inhibitors and angiotensin receptor blockers (ARBs), which were grouped as renin angiotensin system (RAS) blocker. Lipid-lowering medications included statins and fibrates. Insulin therapy was defined as continuous dispensing of insulin for at least 6 months.

### Genotyping

We genotyped 14 genetic variants (*NOTCH2* rs10923931, *ADAMTS9* rs4607103, *IGF2BP2* rs4402960, *WFS1* rs734312, *CDKAL1* rs7756992, *JAZF1* rs864745, *SLC30A8* rs13266634, *CDKN2A/B* rs10811661, *HHEX* rs7923837, *TCF7L2* rs7903146, *KCNQ1* rs2237892, *KCNJ11* rs5219, *TSPAN8/LGR5* rs7961581, *HNF1B* rs4430796) associated with T2D and beta-cell dysfunction in multiple populations including Chinese. We did not test for associations for all tagging single nucleotide polymorphisms (SNPs) of the respective genes. Genotyping on genomic DNA was performed either at deCODE Genetics using the Centaurus (Nanogen) platform or at the McGill University and Genome Quebec Innovation Centre using the Sequenom MassARRAY platform (San Diego, CA, USA). All SNPs were in Hardy-Weinberg equilibrium (*P*>0.01) in control cohorts using the exact test implemented in PLINK [47]. The overall genotype call rates were >95% and the minor allele frequencies (MAF) in normal controls were comparable with the HapMap CHB data.

### Computation of Combined Genetic Score (CGS)

We selected SNPs with alleles associated with T2D consistent with the literature and *P* values <0.05 to calculate the CGS using two approaches. In the simple count method, we assumed similar effect sizes for each SNP and assigned each subject an unweighted CGS based on the sum of risk alleles. In the weighted method, the number of risk allele for each SNP was multiplied by a weight derived from its relative effect size (*β*-coefficient) estimated in the present study. In this combined cohort, 2.7% had missing genotypes which were imputed by the average-risk allele at each SNP and the CGS for each individual was then rounded to the nearest value.

### Statistical Analysis

All statistical analyses were performed using the Statistical Package for Social Sciences for Windows version 15 (SPSS, Chicago, IL, USA), PLINK v1.07 (http://pngu.mgh.harvard.edu/purcell/plink/), and R 2.15.1 (http://www.r-project.org/) unless not specified otherwise. A 2-tailed *P* value <0.05 was considered significant.

We estimated the study power using Quanto. Assuming an additive model with the at-risk allele frequencies ranging between 5–50% for the variant, the sample size of the case-control cohort at hand would provide >75% power to detect the association with T2D risk at α level of 0.05, assuming prevalence of 0.1 and an odds ratio of 1.2. In addition, assuming that the total explained QTL variances ranges from 0.1 to 1%, the current sample size in the quantitative trait analysis would provide us 68–99% power to detect the association at α level of 0.05.

Data are expressed as percentage, mean±SD or median (interquartile range), as appropriate. Continuous variables (FPI, HOMA-IR and HOMA-β) were log transformed to approximate normal distribution. Each trait was winsorized separately within adolescent and adult cohorts by replacing extreme values with 4 standard deviations from the mean. Less than 0.2% of data were replaced.

We conducted logistic regression analysis with adjustments for sex, age and BMI to compare the genotypes frequencies and CGS between T2D cases and healthy controls under a log additive model. Odd ratios (ORs) with 95% confidence intervals (CIs) were presented. The difference in distributions of CGS between T2D patients and healthy controls were compared by Student’s *t*-test. Multiple testings were corrected by permutations for 10,000 times.

Associations of glucose-related quantitative traits and clinical features with individual SNPs and/or categorized CGS (according to the quartiles of CGS) were tested by linear and logistic regression analysis for continuous and categorical variables, respectively. The covariates included in the regression analyses were selected based on our previous studies [48], [49], [50], [51]: we adjusted for sex, age, BMI and “study cohort” (a dummy variable coded as 0 for adult controls and 1 for adolescent controls) in glucose-related quantitative traits analysis; analysis for age at diagnosis (AAD) was adjusted for sex, BMI and HbA_1c;_ analysis for BMI, WC and central obesity were adjusted for sex and age_;_ analysis for HbA_1c_ was adjusted for sex, age and BMI_;_ analysis for the proportion of insulin therapy at baseline was adjusted for sex, age, smoking status, HbA_1c_, eGFR at baseline and drug usage (lipid lowering, blood pressure lowering, RAS inhibitors and oral glucose lowering drugs). The genetic effects on quantitative traits were presented by either β±SE estimated from the linear regression model or the marginal mean (95% CIs) estimated from general linear model adjusted for covariates, categorized by the number of risk alleles.

In the sub-phenotype analysis for T2D risk, multiplicative interaction between overweight (BMI≥25 kg/m^2^ vs BMI<25 kg/m^2^) and CGS was tested by logistic regression analysis including the main and product interaction terms of overweight and CGS. Cochran’s *Q* statistic (*P*<0.05) and *I^2^* index were used to assess heterogeneity of ORs between subgroups.

To evaluate the discriminative power of the prediction model on T2D risk, we calculated the area under the receiver operating characteristic (ROC) curve, denoted area under curve (AUC) based on the predicted risks for each individual obtained from the logistic regression analysis. Three different prediction models were considered: 1) including clinical variables (sex, age and BMI) only; 2) including unweighted or weighted CGS only; and 3) including both clinical variables and CGS. The AUC can vary from 0.5 (no discrimination) to one (prefect discrimination). Furthermore, the contribution of CGS was assessed by the net reclassification improvement (NRI) method which evaluates the proportion of subjects moving accurately or inaccurately from one risk category to another after adding CGS into the model. Typically, NRI analysis is applied in studies with prospective follow-up. In order to apply NRI analysis in our case-control study, we adopted the approach proposed by Pencina et al [52]. We included the term of log[*ρ/*(1−*ρ*)×*n_control/_n_case_*] to the intercept of logistic regression model to adjust for predicted risk with prevalence *ρ*.

## Results

### Single SNP Association for T2D Risk, Age of Diagnosis and Glucose-related Traits

We genotyped 14 SNPs relating to beta-cell function in 5882 T2D patients and 2569 healthy controls. Of these, 8 SNPs including *IGF2BP2* rs4402960, *WFS1* rs734312, *CDKAL1* rs7756992, *SLC30A8* rs13266634, *CDKN2A/B* rs10811661, *HHEX* rs7923837, *TCF7L2* rs7903146 and *KCNQ1* rs2237892 were consistently and significantly associated with T2D after adjusting for sex, age and BMI (OR = 1.14–2.09, 8.5×10^−18^<*P*<8.5×10^−3^) (Table 2). The association of *KCNQ1* rs2237892 was the strongest (*P*<8.5×10^−18^) while *TCF7L2* rs7903146 showed the largest effect (OR [95% CI] = 2.09 [1.63–2.69]), albeit with rare allele frequency (0.034 in T2D patients; 0.019 in healthy controls). Nominal associations were found at *NOTCH2* rs10923931, *JAZF1* rs864745, *KCNJ11* rs5219, and *HNF1B* rs4430796 with ORs ranging from 1.07 to 1.24 (0.0516<*P*<0.0816) (Table 2), but not for *ADAMTS9* rs4607103 and *TSPAN8/LGR5* rs7961581. All significant SNPs except *WFS1* rs734312 remained statistically significant after correcting for multiple comparisons (Table 2). Among the 14 SNPs examined in this analysis, the probability that 12 or more SNPs (*P*≤0.1) would come up with effect estimates that point in the same direction as previous reports is 6.5×10^−3^ based on the binomial distribution.

**Table 2:** Associations of single nucleotide polymorphisms (SNPs) of replicated genetic loci with type 2 diabetes and age at diagnosis in Chinese populations.

				Risk allele frequency		Type 2 Diabetes in 5882cases and 2569 controls			Age at diagnosis in 5882 cases		
Chr	SNP	Gene	Risk/non-riskallele	Cases	Controls	OR (95% CI)	*P*	*P_permutation_*	*β* (SE)	*P*	*P_permutation_*
1	rs10923931	*NOTCH2*	T/G	0.037	0.029	1.24 (1.00–1.53)	0.0516	0.5293	0.838 (0.627)	0.1817	0.9403
3	rs4607103	*ADAMTS9*	C/T	0.680	0.691	0.97 (0.89–1.05)	0.4293	0.9996	0.346 (0.253)	0.1719	0.9308
3	rs4402960	*IGF2BP2*	T/G	0.251	0.230	1.16 (1.06–1.27)	8.5×10^−4^	0.0132	−0.172 (0.270)	0.5250	1.0000
4	rs734312	*WFS1*	A/G	0.817	0.799	1.14 (1.03–1.25)	8.5×10^−3^	0.1131	−0.269 (0.306)	0.3792	0.9979
6	rs7756992	*CDKAL1*	G/A	0.506	0.459	1.22 (1.14–1.32)	1.0×10^−7^	1.0×10^−4^	−0.456 (0.231)	0.0482	0.5054
7	rs864745	*JAZF1*	A/G	0.783	0.771	1.08 (0.99–1.19)	0.0816	0.6958	−0.350 (0.288)	0.2242	0.9710
8	rs13266634	*SLC30A8*	C/T	0.568	0.527	1.22 (1.13–1.32)	2.2×10^−7^	1.0×10^−4^	−0.708 (0.236)	2.8×10^−3^	0.0364
9	rs10811661	*CDKN2A/B*	T/C	0.618	0.579	1.21 (1.12–1.31)	9.7×10^−7^	1.0×10^−4^	−0.382 (0.242)	0.1142	0.8226
10	rs1111875	*HHEX*	G/A	0.302	0.274	1.22 (1.12–1.32)	2.9×10^−6^	1.0×10^−4^	−0.373 (0.256)	0.1452	0.8905
10	rs7903146	*TCF7L2*	T/C	0.034	0.019	2.09 (1.63–2.69)	9.6×10^−9^	1.0×10^−4^	−0.664 (0.648)	0.3060	0.9936
11	rs2237892	*KCNQ1*	C/T	0.719	0.656	1.45 (1.33–1.58)	8.5×10^−18^	1.0×10^−4^	−0.911 (0.277)	1.0×10^−3^	0.0132
11	rs5219	*KCNJ11*	T/C	0.342	0.325	1.07 (0.99–1.16)	0.0772	0.6739	−0.095 (0.247)	0.7006	1.0000
12	rs7961581	*TSPAN8/LGR5*	C/T	0.228	0.229	0.98 (0.89–1.07)	0.6187	1.0000	0.087 (0.278)	0.7533	1.0000
17	rs4430796	*HNF1B*	G/A	0.267	0.254	1.09 (1.00–1.18)	0.0617	0.5888	−0.125 (0.267)	0.6393	1.0000

The ORs (95% CIs) and *P* values for type 2 diabetes were calculated using logistic regression analysis adjusted for sex, age and BMI assuming an additive genetic model in 5882 cases and 2569 controls. The *βs* (SEs) and *P* values for age at diagnosis were calculated using linear regression analysis adjusted for sex, BMI and HbA_1c_ assuming an additive genetic model in 5882 cases. ORs (95%CIs) and *βs* (SEs) were reported with respect to the risk allele described in literature.

Next, we examined the effects of genetic variants on AAD in T2D patients and glucose-related quantitative traits in healthy adolescents and adults. The reported T2D risk alleles for three SNPs (*CDKAL1* rs7756992, *SLC30A8* rs13266634 and *KCNQ1* rs2237892) were associated with younger AAD (1.0×10^−3^<*P*<0.0482) (Table 2). Elevated FPG and reduced beta-cell function (assessed by HOMA-β) were also associated with T2D risk alleles of *CDKN2A/B* rs10811661 (*β*±S.E. = 0.036±0.013, *P* = 5.5×10^−3^) and *SLC30A8* rs13266634 (*β*±S.E. = −0.042±0.021, *P* = 0.0438), respectively (Table S1).

### Combined Genetic Effect on T2D Risk, Glucose-related Traits in Healthy Adolescents and Adults, as well as Clinical Features in T2D Patients

**Figure 1:**
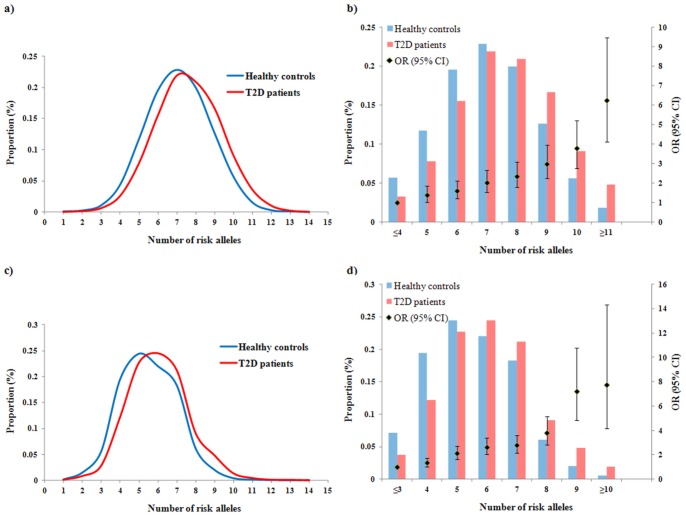
Distributions and effects of unweighted (a–b) and weighted (c–d) CGSs on T2D risk.

We further investigated the joint genetic effect on T2D risk. Figures 1a and 1c showed the distributions of unweighted and weighted CGS between T2D patients and healthy controls, respectively. For both CGSs, a greater proportion of T2D patients carried a higher number of risk alleles than healthy controls. Patients with T2D had more risk alleles (mean±SD = 7.60±1.69 and 6.03±1.59 for unweighted and weighted CGS) than healthy controls (mean±SD = 7.08±1.69 and 5.53±1.52 for unweighted and weighted CGS, respectively) (*P* of *t*-test = 4.0×10^−37^ and 6.6×10^−42^ for unweighted and weighted CGS, respectively). In multivariate logistic regression analysis, each additional risk allele resulted in increasing odds of T2D by 1.24 (95% CI = 1.20–1.28, *P* = 2.2×10^−40^) and 1.29 (95% CI = 1.25–1.34, *P* = 2.2×10^−45^) for unweighted and weighted CGS, respectively (Figure 1b and 1d). Subjects carrying ≥11 risk alleles in unweighted CGS had an OR of 6.25 (95% CI = 4.13–9.47, *P* = 4.8×10^−18^) compared to those carrying ≤4 risk alleles (Figure 1b). Similarly, subjects carrying ≥10 risk alleles in weighted CGS had an OR of 7.75 (95% CI = 4.18–14.36, *P* = 7.9×10^−11^) compared to those carrying ≤3 risk alleles (Figure 1d).

To explore the effect of CGS on glucose-related traits and clinical features, we divided all participants into 4 groups by quartiles of CGS. In healthy adolescents and adults, increasing number of risk alleles was moderately associated with lower HOMA-β (*β*±SE = −0.031±0.015, *P_unweighted_* CGS = 0.0339; *β*±SE = −0.029±0.014, *P_weighted_* CGS = 0.0422). A trend was observed for higher FPG using the unweighted CGS (*β*±SE = 0.018±0.009, *P_unweighted_* CGS = 0.0502) but was no longer significant for the weighted CGS (*β*±SE = 0.012±0.009, *P_weighted_* CGS = 0.1986) (Figure S1a–d). No association was observed for any traits with both unweighted and weighted CGS after Bonferroni correction. In patients with T2D, those with more risk alleles were leaner (BMI: *P_unweighted CGS_* = 4.4×10^−9^, *P_weighted CGS_* = 2.3×10^−10^; WC: *P_unweighted CGS_* = 5.0×10^−6^ and 2.7×10^−4^ for male and female, *P_weighted CGS_* = 4.5×10^−7^ and 9.7×10^−5^ for male and female; Central obesity: *P_unweighted CGS_* = 1.5×10^−4^, *P_weighted CGS_* = 6.9×10^−5^), had younger AAD (*P_unweighted CGS_* = 9.4×10^−7^, *P_weighted CGS_* = 5.6×10^−7^), higher rates of positive family history of T2D (*P_unweighted CGS_* = 0.0261, *P_weighted CGS_* = 0.0218) and were more likely to be treated with insulin at time of recruitment (*P_unweighted CGS_* = 0.0332, *P_weighted CGS_* = 0.0249) (Table 3).

**Table 3:** Clinical features of subjects with type 2 diabetes (T2D) in each quartile of the unweighted (upper panel) and weighted (lower panel) combined genetic score (CGS), respectively.

	CGS quartile				
Clinical features	Q1	Q2	Q3	Q4	*P*
*Unweighted CGS*					
Genetic score: median (min – max)	6 (2–6)	7 (7–7)	8 (8–9)	10 (10–14)	**–**
N	1567	1290	2210	815	–
Sex (male %)	44.61%	45.58%	46.06%	45.64%	0.8504
Age (year)	57.63±13.09	56.85±13.18	56.23±13.36	56.33±13.5	1.9×10^−3^
Age at diagnosis (year)	50.66±12.69	50.04±12.53	49.13±12.54	48.62±12.81	9.4×10^−7^
Duration of T2D (year)	6.97±6.71	6.81±6.63	7.1±6.59	7.72±6.94	0.0276
First degree family history of T2D (%)	39.12%	39.61%	42.4%	42.45%	0.0261
Body mass index (kg/m^2^)	25.43±3.99	25.2±4.02	24.95±3.83	24.52±3.85	4.4×10^−9^
Waist circumference (cm)					
male	89.62±9.81	88.4±9.59	88.07±9.51	86.84±9.33	5.0×10^−6^
female	84.41±10.14	84.32±10.23	82.95±9.52	82.71±9.8	2.7×10^−4^
Central obesity (%)	58.69%	55.33%	53.47%	50.75%	1.5×10^−4^
HbA_1c_ (%)	7.76±1.82	7.67±1.81	7.7±1.8	7.9±1.81	0.5295
Insulin therapy at baseline (%)	20.61%	20.39%	21.54%	23.31%	0.0332
*Weighted CGS*					
Genetic score: median (min – max)	5 (1–5)	6 (6–6)	7 (7–7)	8 (8–13)	–
N	2268	1440	1247	927	–
Sex (male %)	44.84%	45.69%	45.71%	46.6%	0.827
Age (year)	57.5±13.23	56.39±13.02	56.09±13.55	56.37±13.37	5.2×10^−3^
Age at diagnosis (year)	50.62±12.73	49.41±12.31	48.94±12.65	48.7±12.74	5.6×10^−7^
Duration of T2D (year)	6.88±6.58	6.98±6.66	7.15±6.83	7.67±6.77	0.0053
First degree family history of T2D (%)	39.15%	41.25%	42.18%	43.04%	0.0218
Body mass index (kg/m^2^)	25.36±4.01	25.17±3.97	24.92±3.87	24.43±3.66	2.3×10^−10^
Waist circumference (cm)					
male	89.13±9.89	89.08±9.4	87.61±9.31	86.55±9.41	4.5×10^−7^
female	84.36±10.07	83.84±10.01	82.77±9.88	82.51±9.2	9.7×10^−5^
Central obesity (%)	57.17%	57.33%	51.21%	50.49%	6.9×10^−5^
HbA_1c_ (%)	7.73±1.83	7.69±1.79	7.7±1.76	7.87±1.86	0.3552
Insulin therapy at baseline (%)	20.24%	21.67%	21.17%	23.41%	0.0249

Data are shown as N, %, mean±SD or median (minimum to maximum). Between-group comparisons of clinical characteristics were performed by χ^2^ test or logistic regression analysis for categorical variables, and one-way ANOVA or linear regression analysis for continuous variables, as appropriate. Analysis for age at diagnosis was adjusted for sex, body mass index (BMI) and HbA_1c_. Analysis for BMI and central obesity were adjusted for sex and age. Analysis for waist circumference (stratified by sex) was adjusted for age. Analysis for HbA_1c_ was adjusted for sex, age and BMI. Analysis for insulin therapy was adjusted for sex, age, smoking status, HbA_1c_, baseline eGFR and drug usages (lipid lowering, blood pressure anti-hypertensive, ACE inhibitor and oral glucose lowering). Q1, quartile 1; Q2, quartile 2; Q3, quartile 3; Q4, quartile 4; T2D, type 2 diabetes.

### Sub-phenotype Analysis on T2D Risk Stratified by Overweight and Non-overweight Subjects

To test for the heterogeneity of T2D risk with CGS between overweight and non- overweight subjects, we stratified the subjects into two groups: overweight group defined as BMI≥25 kg/m^2^ and non-overweight group defined as BMI<25 kg/m^2^. There were strong associations of CGS with T2D risk in both groups for unweighted and weighted CGS (*P*<0.0001) (Figure S2). In the non-overweight group, the OR (95% CI) per copy of risk allele (1.26 (1.21–1.31)) increased exponentially across the counts of unweighted CGS, and also more steeply compared to the OR in the overweight group (1.17 (1.10–1.24) per copy of risk allele, *P_unweighted = _*0.0312 and *I* ^2^ = 0.7846 in heterogeneity test of OR). We did not detect any interaction between CGS and overweight/non-overweight groups for T2D risk (*P*>0.05).

### Predictive Power of CGS for T2D Risk

We assessed discrimination and reclassification to evaluate the contribution of CGS for predicting T2D risk. Firstly, AUC was used to assess the discriminatory power of the model with and without inclusion of CGS on top of clinical variables (sex, age and BMI). The AUC was 0.75 (95% CI = 0.74–0.76) for the model incorporating clinical variable alone, then increased marginally by 0.02 when both clinical variables and CGS were included (Figure S3 and Table S2).

To directly compare the clinical impact of models with and without CGS, net reclassification improvement (NRI) was computed to indicate the proportion of subjects reclassified correctly (NRI>0) or incorrectly (NRI<0) into various risk categories. We conducted the analysis separately for T2D patients and healthy controls and stratified them into five risk categories (<5%, 5 to <10%, 10 to <15%, 15 to <20% and ≥20%) based on the clinical variables. When we included the unweighted/weighted CGS in addition to the clinical variables, 22.0%/22.2% of T2D patients were correctly reclassified to higher risk category and 16.6%/17.8% incorrectly reclassified to lower risk category. Similarly, 15.4%/17.1% of healthy controls correctly moved down to lower risk category and 9.8%/10.1% incorrectly moved up to higher risk category. These reclassification rates gave an estimated NRI of 11.0% (95% CI = 7.5–14.5, *P*<0.001) and 11.4% (95% CI = 7.7–15.1, *P*<0.001) by including the unweighted and weighted CGS, respectively (Table 4 and Table 5).

**Table 4:** Reclassification of predicted risk with the addition of unweighted combined genetic score (CGS) including 8 variants (*P*<0.05) in T2D subjects (upper panel) and healthy controls (lower panel).

	Reclassified predicted risk (with CGS)					% (N) of subjects reclassified with		
Predicted risk (without CGS)	<5%	5 to <10%	10 to <15%	15 to <20%	≥20%	increased risk	decreased risk	Net correctly reclassified (%)
**T2D patients (N = 5820)**								
** <5%**	248	106	0	0	0	22.0%	16.6%	5.38%
** 5 to <10%**	129	679	290	73	0	(1280)	(967)	
** 10 to <15%**	0	278	500	316	134			
** 15 to <20%**	0	34	240	307	361			
** ≥20%**	0	0	61	225	1839			
**Healthy controls (N = 2560)**								
** <5%**	1151	41	0	0	0	9.8%	15.4%	5.63%
** 5 to <10%**	100	224	64	5	0	(250)	(394)	
** 10 to <15%**	0	118	132	68	13			
** 15 to <20%**	0	11	79	80	59			
** ≥20%**	0	0	20	66	329			
**Net reclassification improvement (95% CI)**								11.0 (7.5–14.5)
								*P*<0.001

CGS: combined genetic score. Each cell refers to the number of subjects in the predicted risk categories. Subjects with higher predicted risk were more likely to be classified as cases. Similarly, subjects with lower predicted risk were more likely to be classified as controls. T2D subjects and healthy controls classified in the shaded cells indicated that they were correctly reclassified to higher and lower risk categories, respectively. The total number of subjects reclassified is 2,891 and the improvement classification rates are 5.38% and 5.63% for T2D subjects and healthy controls, respectively with a total improvement rate of 11.0% (5.38% +5.63%).

**Table 5:** Reclassification of predicted risk with the addition of weighted combined genetic score (CGS) including 8 variants (*P*<0.05) in T2D subjects (upper panel) and healthy controls (lower panel).

	Reclassified predicted risk (with CGS)					% (N) of subjects reclassified with		
Predicted risk (without CGS)	<5%	5 to <10%	10 to <15%	15 to <20%	≥20%	increased risk	decreased risk	Net correctly reclassified (%)
**T2D patients (N = 5820)**								
** <5%**	252	93	9	0	0	22.2%	17.8%	4.36%
** 5 to <10%**	139	678	250	87	17	(1290)	(1036)	
** 10 to <15%**	0	307	460	292	169			
** 15 to <20%**	0	25	261	283	373			
** ≥20%**	0	0	69	235	1821			
**Healthy controls (N = 2560)**								
** <5%**	1151	41	0	0	0	10.1%	17.1%	6.99%
** 5 to <10%**	114	207	64	8	0	(258)	(437)	
** 10 to <15%**	1	128	122	66	14			
** 15 to <20%**	0	19	73	72	65			
** ≥20%**	0	0	34	68	313			
**Net reclassification improvement (95% CI)**								11.4 (7.7–15.1)
								*P*<0.001

CGS: combined genetic score. Each cell refers to the number of subjects in the predicted risk categories. Subjects with higher predicted risk were more likely to be classified as cases. Similarly, subjects with lower predicted risk were more likely to be classified as controls. T2D subjects and healthy controls classified in the shaded cells indicated that they were correctly reclassified to higher and lower risk categories, respectively. The total number of subjects reclassified is 3,021 and the improvement classification rates are 4.36% and 6.99% for T2D subjects and healthy controls, respectively with a total improvement rate of 11.4% (4.36% +6.99%).

To compare the predictive power between CGS based on 8 SNPs with *P*<0.05 and CGS based on 12 SNPs with *P*<0.1, ROC analysis and calculation of NRI were repeated using CGS based on 12 SNPs. However, the additional 4 SNPs with nominal significance (0.05<P<0.1) did not improve the discriminatory power (Figure S4 and Table S3 for ROC analysis, Table S4 and Table S5 for NRI calculation).

## Discussion

Genome-wide association studies have so far identified more than 70 novel loci for T2D with modest effects (OR = 1.06–1.40) [53]. Most of these associations had been replicated in European and Asian populations [23], [24], [25], [26], [27], [29], [33], [34], [35], [54], [55], [56], [57]. In our previous meta-analysis, we reported both individual and joint effects of 7 SNPs in *IGF2BP2*, *CDKAL1*, *SLC30A8*, *CDKN2A/B*, *HHEX*, *TCF7L2* and *FTO* on T2D risk in Chinese and Korean populations [32]. Here we further genotyped 14 loci (6 of which were included in the previous study) relating to impaired beta-cell function in a larger cohort consisting of 5882 T2D patients and 2569 healthy controls in the Chinese population.

Consistent with earlier studies in Caucasians [13], [14], [15], [16], [18], [19], [20], [58], we replicated the associations of T2D with 8 SNPs in *IGF2BP2*, *WFS1*, *CDKAL1*, *SLC30A8*, *CDKN2A/B*, *HHEX*, *TCF7L2* and *KCNQ1* (*P*<0.05), as well as trends of associations in *NOTCH2*, *JAZF1*, *KCNJ11* and *HNF1B* (0.05<*P*<0.1). Their moderate effect sizes (ORs = 1.07–1.45) are similar to those of other studies [13], [14], [15], [16], [18], [19], [20], [58], except for *TCF7L2* which had a high OR of 2.09 albeit a low MAF in Chinese population (0.026 vs 0.279 for Hapmap CHB and CEU, respectively).

To better understand the mechanisms of genetic factors involved in the pathogenesis of T2D, glucose homeostasis and beta-cell function, the Meta-Analyses of Glucose and Insulin-related traits Consortium (MAGIC) has conducted a meta-analysis of GWAS on glycemic quantitative traits [59], [60], [61]. Although most of the susceptibility loci were shown to affect insulin secretion and beta-cell function [62], we only observed the effect of variants in *SLC30A8* and *CDKN2A/B* on glucose-related traits in our Chinese populations. While our findings were concordant with that reported by Wu *et al*. [56] and Ruchat *et al*. [63], there were also negative reports in other Asian studies [23], [24], [34]. Interestingly, Hu *et al*. [34] reported that the C-allele of rs13266634 in *SLC30A8* was associated with higher FPG, in our study, the same allele was associated with lower beta-cell function. On the other hand, while the T-allele of rs10811661 in *CDKN2A/B* was reported to be associated with reduced 2-hour insulin [34] and HOMA-β levels [23], we found an association with increased FPG level. Although association of reduced beta cell function with *TSPAN8/LGR5* had been reported [22], we were not able to confirm these findings in our Chinese population. These discrepant findings might be due to differences in genomic structures, sample size, variability of outcome measures, effect sizes, ethnicity, cultural and environmental factors. For example, we observed remarkable differences of the allele frequencies for most of the examined SNPs between the Chinese and European populations (Table S6). Besides, our sample size only had 66% and 59% power to detect T2D risk with an OR of 1.09 for *ADAMTS9* and *TSPAN8/LGR5* at significance level of 0.05, respectively, thus a larger cohort will be needed to confirm these associations.

Early studies suggested the predictive power of genetic markers for T2D can be improved by using a cumulative number of risk alleles [26], [31], [35]. Therefore, we constructed two CGSs, unweighted and weighted, based on 8 susceptibility loci relating to beta-cell function. Compared to carriers with ≤4 (≤3) alleles, each additional allele increased the odds of T2D by 1.24-fold (1.29-fold) for unweighted (weighted) CGS. These values were similar to those reported by Hoek *et al*. [33], Miyake *et al*. [35], and Wu *et al*. [56], despite differences in ethnicity, study design and selection of genetic variants. Quartile analyses of CGS further showed that subjects carrying more risk alleles were less obese, had earlier AAD, a trend of higher FPG and lower HOMB-β levels, and were more likely to be insulin-treated. Taken together, our findings and those of others [24], [64], strongly support the notion that these genetic variants increase T2D risk through pancreatic beta-cell dysfunction.

The utility of genetic markers in the prediction of common diseases can be substantially improved by identifying the interactions between genetic and environmental factors. [65]. For instance, Linder *et al*. [66] suggested that the association between impaired glucose tolerance and genetic risk score was modulated by gender, obesity status and insulin sensitivity. To better understand the underlying causal pathways, we examined for possible heterogeneity of T2D risk with CGS between overweight and non-overweight subjects. We observed that the risk association in the non-overweight group showed larger effect size than that in the overweight group (OR 1.26 vs 1.17). Our findings echoed similar findings in a Japanese study where the CGS predicted T2D in non-obese but not obese/overweight subjects [27]. Similarly, the risk association of insulin resistance related loci with T2D risk showed larger effect size in obese individuals while that of insulin secretion related loci showed larger effect in non-obese individuals [67]. In this analysis, we selected 8 SNPs implicated in beta-cell function, which might explain the larger effect of the CGS in the non-overweight subjects.

We used two different approaches, discrimination and reclassification to evaluate whether the addition of CGS improved the prediction of T2D risk above and beyond clinical variables. In ROC analysis, AUC was commonly used to measure the discriminatory ability of a model correctly classifying subjects with or without disease. In many studies, the additional contribution attributed to genetic variants detected by ROC curve has been minimal [25], [26], [29], [33], [34], [35]. Consistent with this, our results showed that the addition of genetic information only increased the AUC by 2% for both unweighted and weighted CGSs, despite the strong and independent association of CGSs with T2D in the logistic regression analysis. This might be in part due to the confounding effect of BMI on the association between CGS and T2D and the insensitivity of ROC analysis to small changes in risk. For clinical risk prediction, it is important to evaluate whether a new model can correctly classify individuals into higher or lower risk categories [68]. Recently, Pencina *et al*. introduced a measure named NRI to quantity the degree of correct reclassification [52]. By using this approach, we demonstrated that the addition of genetic information to clinical variables (sex, age and BMI) was significant and provided >11% net reclassification improvement (*P*<0.0001).

To our knowledge, this is the first study confirming the utility of genetic factors for predicting T2D risk using the NRI approach. However, several limitations need to be considered. Firstly, our control cohort consisted of adolescents who might develop diabetes in the future. In our sensitivity analysis, removal of either all subjects in the adolescent cohort or adolescents aged <16 years resulted in similar effect sizes as compared to Table 2 (data not shown). In addition, only a few potential common genetic variants were tested for association with T2D risk in this study. Also, we have not interrogated the gene structure and the possibility of closely linked causal variants, gene-gene and/or gene-environmental interactions, as well as possible ethnic differences in gene expression. More genes and their interactions have to be detected and incorporated into the computation of genetic scores. Thirdly, the results for risk prediction should be interpreted with caution in a case-control study. In general, data from population-based studies is preferred for evaluation of risk prediction models because they incorporate information of true disease prevalence. Hence, we performed the NRI analysis separately among cases and controls, as well as adjusted the case-control intercept using the T2D incidence of 10% in the Chinese population to obtain the meaningful predicted risks from logistic regression model. The representative nature of our cohort and robustness of our analysis was also evident by comparing the odds ratios to that of other cohort studies (OR = 1.00–1.36 for individual SNPs, OR = 1.18–1.20 for CGS) [26], [27], [29]. Finally, our prediction model included the commonly used clinical variables (sex, age and BMI) but did not include the other risk factors for T2D such as blood pressure and lipid profiles. Additional studies are warranted to verify our findings.

In Chinese, the use of a CGS comprising 8 reported susceptibility loci, modestly but significantly, improved the predictive ability for T2D risk above and beyond that attributed to clinical variables (sex, age and BMI). The discovery of additional variants through large-scale GWAS and whole genome sequencing will further improve the robustness of these predictive tools to identify high risk subjects for early intervention, in addition to providing novel pathways for personalized care.

## Supporting Information

Figure S1 **Per-alleic effects of unweighted (red) and weighted (blue) combined genetic scores on glucose related quantitative traits ((a) fasting plasma glucose, (b) fasting plasma insulin, (c) HOMA-IR and (d) HOMA-β) in healthy adolescents and adults.**
(DOCX)

Figure S2 **Odds ratios for T2D risk associated with a) unweighted GCS and b) weighted CGS in cases vs. controls, stratified by BMI (BMI<25 kg/m^2^ and BMI≥25 kg/m^2^).**
(DOCX)

Figure S3 **ROC curves for discrimination between T2D patients and healthy controls based on 3 models.** Model 1 includes conventional risk factors (sex, age and BMI). Model 2 includes (unweighted or weighted) combined genetic scores based on 8 variants (*P*<0.05). Model 3 includes both.
(DOCX)

Figure S4 **ROC curves for discrimination between T2D patients and healthy controls based on 3 models.** Model 1 includes conventional risk factors (sex, age and BMI). Model 2 includes (unweighted or weighted) combined genetic scores based on 12 variants (*P*<0.1). Model 3 includes both.
(DOCX)

Table S1 Associations of SNPs with glucose related quantitative traits in healthy adolescents and adults.
(DOCX)

Table S2 Multivariate logistic regression and AUC for T2D based on 3 models. Model 1 includes conventional risk factors (sex, age and BMI). Model 2 includes (unweighted or weighted) combined genetic scores based on 8 variants (*P*<0.05). Model 3 includes both.
(DOCX)

Table S3 Multivariate logistic regression and AUC for T2D based on 3 models. Model 1 includes conventional risk factors (sex, age and BMI). Model 2 includes (unweighted or weighted) combined genetic scores based on 12 variants (*P*<0.1). Model 3 includes both.
(DOCX)

Table S4 Reclassification of predicted risk with the addition of unweighted combined genetic score (CGS) based on 12 variants (*P*<0.1) in T2D subjects (upper panel) and healthy controls (lower panel).
(DOCX)

Table S5 Reclassification of predicted risk with the addition of weighted combined genetic score (CGS) based on 12 variants (*P*<0.1) in T2D subjects (upper panel) and healthy controls (lower panel).
(DOCX)

Table S6 Comparison of at-risk allele frequencies between the Chinese and European populations using the HapMap data.
(DOCX)
