# Supplementary material for: Use of Net Reclassification Improvement (NRI) Method Confirms The Utility of Combined Genetic Risk Score to Predict Type 2 Diabetes
Source: PLoS One. 2013 Dec 20;8(12):e83093. doi: 10.1371/journal.pone.0083093 (PMC3869744; doi:10.1371/journal.pone.0083093)
Supplement: Figure S1 — Per-alleic effects of unweighted (red) and weighted (blue) combined genetic scores on glucose related quantitative traits ((a) fasting plasma glucose, (b) fasting plasma insulin, (c) HOMA-IR and (d) HOMA-β) in healthy adolescents and adults. (DOCX) [file pone.0083093.s001.docx]

**Figure S1. Per-alleic effects of unweighted (red) and weighted (blue) combined genetic scores on glucose related quantitative traits ((a) fasting plasma glucose, (b) fasting plasma insulin, (c) HOMA-IR and (d) HOMA-β) in healthy adolescents and adults. There are 950, 587, 839 and 193 subjects in Q1, Q2, Q3 and Q4, respectively for unweighted genetic score. There are 1311, 565, 470 and 223 subjects in Q1, Q2, Q3 and Q4, respectively for weighted genetic score.**

**a) b)**

**c) d)**
