# Supplementary material for: Use of Net Reclassification Improvement (NRI) Method Confirms The Utility of Combined Genetic Risk Score to Predict Type 2 Diabetes
Source: PLoS One. 2013 Dec 20;8(12):e83093. doi: 10.1371/journal.pone.0083093 (PMC3869744; doi:10.1371/journal.pone.0083093)
Supplement: Figure S2 — Odds ratios for T2D risk associated with a) unweighted GCS and b) weighted CGS in cases vs. controls, stratified by BMI (BMI<25 kg/m2 and BMI≥25 kg/m2). (DOCX) [file pone.0083093.s002.docx]

**Figure S2. Odds ratios for T2D risk associated with a) unweighted GCS and b) weighted CGS in cases vs. controls, stratified by BMI (BMI < 25 kg/m^2^ and BMI ≥ 25 kg/m^2^)**

1. **Unweighted CGS b) Weighted CGS**
