# Supplementary material for: Use of Net Reclassification Improvement (NRI) Method Confirms The Utility of Combined Genetic Risk Score to Predict Type 2 Diabetes
Source: PLoS One. 2013 Dec 20;8(12):e83093. doi: 10.1371/journal.pone.0083093 (PMC3869744; doi:10.1371/journal.pone.0083093)
Supplement: Figure S4 — ROC curves for discrimination between T2D patients and healthy controls based on 3 models. Model 1 includes conventional risk factors (sex, age and BMI). Model 2 includes (unweighted or weighted) combined genetic scores based on 12 variants (P<0.1). Model 3 includes both. (DOCX) [file pone.0083093.s004.docx]

**Figure S4. ROC curves for discrimination between T2D patients and healthy controls based on 3 models. Model 1 includes conventional risk factors (sex, age and BMI). Model 2 includes (unweighted or weighted) combined genetic scores based on 12 variants (*P* < 0.1). Model 3 includes both.**

**a) Unweighted CGS b) Weighted CGS**
