# Supplementary material for: Use of Net Reclassification Improvement (NRI) Method Confirms The Utility of Combined Genetic Risk Score to Predict Type 2 Diabetes
Source: PLoS One. 2013 Dec 20;8(12):e83093. doi: 10.1371/journal.pone.0083093 (PMC3869744; doi:10.1371/journal.pone.0083093)
Supplement: Table S1 — Associations of SNPs with glucose related quantitative traits in healthy adolescents and adults. (DOCX) [file pone.0083093.s005.docx]

**Table S1. Associations of SNPs with glucose related quantitative traits in healthy adolescents and adults.**

|  |  |  |  | **Fasting plasma glucose** | | **Fasting plasma insulin** | | **HOMA-IR** | | **HOMA-β** | |
| --- | --- | --- | --- | --- | --- | --- | --- | --- | --- | --- | --- |
| **Chr** | **SNP** | **Gene** | **T2D-risk allele** | ***β* ± SE** | ***P*** | ***β* ± SE** | ***P*** | ***β* ± SE** | ***P*** | ***β* ± SE** | ***P*** |
| 1 | rs10923931 | *NOTCH2* | T | -0.009 ± 0.038 | 0.8201 | 0.015 ± 0.052 | 0.7805 | 0.022 ± 0.058 | 0.7058 | 0.025 ± 0.059 | 0.6685 |
| 3 | rs4607103 | *ADAMTS9* | C | 0.005 ± 0.014 | 0.7252 | 0.012 ± 0.020 | 0.5465 | 0.013 ± 0.022 | 0.5660 | 0.003 ± 0.023 | 0.8986 |
| 3 | rs4402960 | *IGF2BP2* | T | -0.010 ± 0.015 | 0.5285 | 0.026 ± 0.021 | 0.2117 | 0.025 ± 0.023 | 0.2817 | 0.032 ± 0.024 | 0.1853 |
| 4 | rs734312 | *WFS1* | A | 0.010 ± 0.016 | 0.5477 | -0.009 ± 0.023 | 0.6897 | -0.007 ± 0.025 | 0.7949 | -0.016 ± 0.026 | 0.5378 |
| 6 | rs7756992 | *CDKAL1* | G | 0.018 ± 0.013 | 0.1631 | -0.007 ± 0.018 | 0.6898 | -0.007 ± 0.020 | 0.7353 | -0.025 ± 0.020 | 0.2209 |
| 7 | rs864745 | *JAZF1* | A | 0.012 ± 0.016 | 0.4317 | 0.024 ± 0.022 | 0.2741 | 0.027 ± 0.024 | 0.2741 | 0.009 ± 0.025 | 0.7220 |
| 8 | rs13266634 | *SLC30A8* | C | 0.016 ± 0.013 | 0.2173 | -0.027 ± 0.018 | 0.1445 | -0.028 ± 0.020 | 0.1754 | -0.042 ± 0.021 | 0.0438 |
| 9 | rs10811661 | *CDKN2A/B* | T | 0.036 ± 0.013 | 0.0055 | 0.000 ± 0.019 | 0.9948 | 0.005 ± 0.020 | 0.8109 | -0.033 ± 0.021 | 0.1127 |
| 10 | rs1111875 | *HHEX* | G | 0.020 ± 0.014 | 0.1711 | 0.008 ± 0.020 | 0.7033 | 0.015 ± 0.022 | 0.4992 | -0.004 ± 0.023 | 0.8480 |
| 10 | rs7903146 | *TCF7L2* | T | -0.028 ± 0.042 | 0.5074 | -0.078 ± 0.059 | 0.1874 | -0.092 ± 0.065 | 0.1589 | -0.043 ± 0.067 | 0.5196 |
| 11 | rs2237892 | *KCNQ1* | C | -0.016 ± 0.014 | 0.2600 | -0.032 ± 0.019 | 0.0981 | -0.040 ± 0.022 | 0.0639 | -0.020 ± 0.022 | 0.3721 |
| 11 | rs5219 | *KCNJ11* | T | -0.005 ± 0.014 | 0.7259 | 0.005 ± 0.019 | 0.7741 | 0.007 ± 0.021 | 0.7293 | 0.013 ± 0.021 | 0.5587 |
| 12 | rs7961581 | *TSPAN8/ LGR5* | C | 0.013 ± 0.015 | 0.3927 | 0.002 ± 0.022 | 0.9306 | 0.005 ± 0.024 | 0.8390 | -0.010 ± 0.024 | 0.6820 |
| 17 | rs4430796 | *HNF1B* | G | -0.002 ± 0.015 | 0.8877 | -0.032 ± 0.021 | 0.1356 | -0.030 ± 0.023 | 0.1975 | -0.027 ± 0.024 | 0.2513 |

The *βs* **±** SEs and *P* values were calculated using linear regression adjusted for sex, age, BMI and study cohort (subject is coded as 0 and 1 for adolescents and adults, respectively) assuming an additive genetic model. *βs* **±** SEs were reported with respect to the T2D-risk allele described in literature.
