# Supplementary material for: Use of Net Reclassification Improvement (NRI) Method Confirms The Utility of Combined Genetic Risk Score to Predict Type 2 Diabetes
Source: PLoS One. 2013 Dec 20;8(12):e83093. doi: 10.1371/journal.pone.0083093 (PMC3869744; doi:10.1371/journal.pone.0083093)
Supplement: Table S2 — Multivariate logistic regression and AUC for T2D based on 3 models. Model 1 includes conventional risk factors (sex, age and BMI). Model 2 includes (unweighted or weighted) combined genetic scores based on 8 variants (P<0.05). Model 3 includes both. (DOCX) [file pone.0083093.s006.docx]

**Table S2. Multivariate logistic regression and AUC for T2D based on 3 models. Model 1 includes conventional risk factors (sex, age and BMI). Model 2 includes (unweighted or weighted) combined genetic scores based on 8 variants (*P* < 0.05). Model 3 includes both.**

|  | **Model 1: clinical variables only** | | **Model 2: CGS only** | | **Model 3: both** | |
| --- | --- | --- | --- | --- | --- | --- |
| **Variable in model** | **OR (95% CI)** | ***P*** | **OR (95% CI)** | ***P*** | **OR (95% CI)** | ***P*** |
| *Unweighted CGS* |  |  |  |  |  |  |
| Sex (Male = 1; Female = 2) | 1.17 (1.05 - 1.30) | 4.0 × 10^-3^ |  |  | 1.17 (1.05 - 1.30) | 4.2 × 10^-3^ |
| Age (years) | 1.03 (1.03 - 1.04) | 7.1 × 10^-121^ |  |  | 1.03 (1.03 - 1.04) | 5.7 × 10^-120^ |
| BMI (kg/m^2^) | 1.23 (1.21 - 1.25) | 2.8 × 10^-150^ |  |  | 1.24 (1.22 - 1.26) | 8.2 × 10^-153^ |
| Combined genetic score |  |  | 1.19 (1.16 - 1.23) | 3.6 × 10^-35^ | 1.24 (1.20 - 1.28) | 8.6 × 10^-40^ |
| **AUC** | 0.747 (0.735 - 0.760) |  | 0.582 (0.569 - 0.595) |  | 0.765 (0.753 - 0.777) |  |
|  |  |  |  |  |  |  |
| *Weighted CGS* |  |  |  |  |  |  |
| Sex (Male = 1; Female = 2) |  |  |  |  | 1.18 (1.06 - 1.31) | 2.9 × 10^-3^ |
| Age (years) |  |  |  |  | 1.03 (1.03 - 1.04) | 3.1 × 10^-119^ |
| BMI (kg/m^2^) |  |  |  |  | 1.24 (1.22 - 1.26) | 8.1 × 10^-154^ |
| Combined genetic score |  |  | 1.24 (1.20 - 1.28) | 3.2 × 10^-41^ | 1.30 (1.26 - 1.35) | 8.2 × 10^-46^ |
| **AUC** |  |  | 0.588 (0.575 - 0.601) |  | 0.769 (0.757 - 0.781) |  |

AUC: area under the receiver operating characteristic (ROC) curve
