# Supplementary material for: Use of Net Reclassification Improvement (NRI) Method Confirms The Utility of Combined Genetic Risk Score to Predict Type 2 Diabetes
Source: PLoS One. 2013 Dec 20;8(12):e83093. doi: 10.1371/journal.pone.0083093 (PMC3869744; doi:10.1371/journal.pone.0083093)
Supplement: Table S5 — Reclassification of predicted risk with the addition of weighted combined genetic score (CGS) based on 12 variants (P<0.1) in T2D subjects (upper panel) and healthy controls (lower panel). (DOCX) [file pone.0083093.s009.docx]

**Table S5. Reclassification of predicted risk with the addition of weighted combined genetic score (CGS) based on 12 variants (*P* < 0.1) in T2D subjects (upper panel) and healthy controls (lower panel).**

|  | **Reclassified predicted risk (with CGS)** | | | | |  | **% (N) of subjects reclassified with** | |  |
| --- | --- | --- | --- | --- | --- | --- | --- | --- | --- |
| **Predicted risk (without CGS)** | **<5%** | **5 to <10%** | **10 to <15%** | **15 to <20%** | **≥20%** |  | **increased risk** | **decreased risk** | **Net correctly reclassified (%)** |
| **T2D patients (N = 5820)** |  |  |  |  |  |  |  |  |  |
| **<5%** | 251 | 99 | 4 | 0 | 0 |  | 22.5% | 17.1% | 5.38% |
| **5 to <10%** | 143 | 648 | 286 | 80 | 14 |  | (1308) | (995) |  |
| **10 to <15%** | 6 | 281 | 481 | 297 | 163 |  |  |  |  |
| **15 to <20%** | 0 | 41 | 244 | 292 | 365 |  |  |  |  |
| **≥20%** | 0 | 4 | 69 | 207 | 1845 |  |  |  |  |
| **Healthy controls (N = 2560)** |  |  |  |  |  |  |  |  |  |
| **<5%** | 1152 | 40 | 0 | 0 | 0 |  | 10.0% | 15.9% | 5.94% |
| **5 to <10%** | 105 | 219 | 61 | 8 | 0 |  | (255) | (407) |  |
| **10 to <15%** | 8 | 110 | 129 | 66 | 18 |  |  |  |  |
| **15 to <20%** | 0 | 22 | 68 | 77 | 62 |  |  |  |  |
| **≥20%** | 0 | 1 | 34 | 59 | 321 |  |  |  |  |
| **Net reclassification improvement (95% CI)** |  |  |  |  |  |  |  |  | 11.3 (8.8 - 13.8) |
|  |  |  |  |  |  |  |  |  | *P* < 0.001 |

CGS: combined genetic score. Each cell refers to the number of subjects in the predicted risk categories. Subjects with higher predicted risk were more likely to be classified as cases. Similarly, subjects with lower predicted risk were more likely to be classified as controls. T2D subjects and healthy controls classified in the shaded cells indicated that they were correctly reclassified to higher and lower risk categories, respectively. The total number of subjects reclassified is 2,965 and the improvement classification rates are 5.38% and 5.94% for T2D subjects and healthy controls, respectively with a total improvement rate of 11.32% (5.38% + 5.94%).
