# Supplementary material for: Use of Net Reclassification Improvement (NRI) Method Confirms The Utility of Combined Genetic Risk Score to Predict Type 2 Diabetes
Source: PLoS One. 2013 Dec 20;8(12):e83093. doi: 10.1371/journal.pone.0083093 (PMC3869744; doi:10.1371/journal.pone.0083093)
Supplement: Table S6 — Comparison of at-risk allele frequencies between the Chinese and European populations using the HapMap data. (DOCX) [file pone.0083093.s010.docx]

**Table S6. Comparison of at-risk allele frequencies between the Chinese and European populations using the HapMap data.**

|  |  |  |  | Allele frequencies | |
| --- | --- | --- | --- | --- | --- |
| Chromosome | SNP | Gene | T2D-risk allele | CEU | CHB |
| 1 | rs10923931 | *NOTCH2* | T | 0.093 | 0.024 |
| 3 | rs4607103 | *ADAMTS9* | C | 0.810 | 0.577 |
| 3 | rs4402960 | *IGF2BP2* | T | 0.296 | 0.238 |
| 4 | rs734312 | *WFS1* | A | 0.650 | 0.869 |
| 6 | rs7756992 | *CDKAL1* | G | 0.279 | 0.476 |
| 7 | rs864745 | *JAZF1* | A | 0.487 | 0.762 |
| 8 | rs13266634 | *SLC30A8* | C | 0.761 | 0.530 |
| 9 | rs10811661 | *CDKN2A/B* | T | 0.801 | 0.577 |
| 10 | rs1111875 | *HHEX* | G | 0.584 | 0.315 |
| 10 | rs7903146 | *TCF7L2* | T | 0.279 | 0.024 |
| 11 | rs2237892 | *KCNQ1* | C | 0.925 | 0.655 |
| 12 | rs7961581 | *TSPAN8/ LGR5* | C | 0.252 | 0.196 |
| 17 | rs4430796 | *HNF1B* | G | 0.509 | 0.250 |
